# LncRNA GHET1 Promotes Hypoxia-Induced Glycolysis, Proliferation, and Invasion in Triple-Negative Breast Cancer Through the Hippo/YAP Signaling Pathway

**DOI:** 10.3389/fcell.2021.643515

**Published:** 2021-04-01

**Authors:** Yu Wang, Shuwei Liu

**Affiliations:** ^1^Research Center for Sectional and Imaging Anatomy Cheeloo College of Medicine, Shandong University, Jinan, China; ^2^School of Basic Medical Sciences, Shandong University, Jinan, China; ^3^Molecular Testing Center, The First Affiliated Hospital of Jinzhou Medical University, Jinzhou, China

**Keywords:** triple-negative breast cancer, hypoxia, Hippo/YAP signaling pathway, lncRNA GHET1, glycolysis

## Abstract

**Objective:**

This study was to assess the specific impacts and mechanism of lncRNA GHET1 in the development of triple-negative breast cancer (TNBC).

**Methods:**

The lncRNA GHET1 expression in TNBC tissues and adjacent healthy tissues was detected by qRT-PCR, and its expression was then measured at the cellular level, including TNBC cells and human normal breast epithelial cell line MCF10A. On the completion of transfection of negative shRNA or lncRNA GHET1 shRNA, the TNBC cells, HCC1937 and MDA-MB-468, were then cultured in a normoxia or hypoxia environment, respectively. 5-Ethynyl-2′-deoxyuridine (EdU) assay, colony formation assay, and transwell assay were applicable to the determination of cell proliferation, cell viability, and invasion in each group, respectively. Reagent kits were used for testing glucose consumption and lactate production levels. HCC1937 cells with knockdown or overexpression of lncRNA GHET1 were injected into the nude mice, followed by the examination of resulting tumor volume and weight. The distribution and expression of Hippo/YAP signaling pathway-related proteins were probed using western blotting.

**Results:**

Highly expressed lncRNA GHET1 in TNBC tissues and cells and induction of lncRNA GHET1 by hypoxia were proved. Knockdown of lncRNA GHET1 significantly reduced proliferation, viability, and invasion of TNBC cells, and decreased glucose consumption and lactate production levels under the hypoxia condition. Furthermore, lncRNA GHET1 knockdown decreased HIF-1α expression in hypoxia and significantly inhibited tumor development *in vivo*. Knockdown of lncRNA GHET1 increased the phosphorylation levels of LATS1 and Yes-associated protein (YAP) to retain YAP within the cytoplasm, while the overexpression of lncRNA GHET1 or hypoxia promoted nuclear translocation of YAP and TNBC development.

**Conclusion:**

LncRNA GHET1 expression can be induced by hypoxia, which leads to excessive activation of the Hippo/YAP signaling pathway, thus promoting TNBC progression.

## Introduction

Breast cancer (BC), the cancer with the highest incidence rate in women, seriously threatens women’s health (Siegel et al., 2019). BC is divided into multiple subtypes according to the surface receptor or gene phenotype. Of all the subtypes, 12–17% are triple-negative breast cancer (TNBC), but its mortality is relatively high (Fan et al., 2014). TNBC does not express a progesterone receptor (PR), a tyrosine kinase receptor-2 (ERB2), and an estrogen receptor (ER), so it is insensitive to standard targeted therapeutic agents for breast cancer and can only be treated with chemotherapy (Biswas and Rao, 2017). In addition, TNBC is clinically characterized by high invasion and susceptibility to local recurrence and distant metastasis, so the prognosis of TNBC is significantly worse compared with other subtypes of breast cancer (Haffty et al., 2006). Hypoxia, which is widespread in tumor tissues, is also an important factor that aggravates the damage caused by tumor cells to the body (Manoochehri Khoshinani et al., 2016). It contributes to the progress of BC and increases the risk of metastasis and mortality (Huang and Zong, 2017). Hypoxia-inducible factor-1α (HIF-1α) is an important metabolic regulator in a variety of tumors, and it mediates the adaptive response to hypoxia in tumors (Semenza, 2012; Singh et al., 2017). After HIF-1α is activated, it can directly induce aerobic glycolysis in many cells, and glycolysis then maintains the survival of cancer cells and promotes cell proliferation, migration, and invasion (Cesi et al., 2017; Huang and Zong, 2017). Therefore, it is very important to explore the mechanism of TNBC development under hypoxia and find reliable biomarkers and therapeutic targets for TNBC.

Long non-coding RNAs (lncRNAs) are defined as non-protein coding transcripts with a length of more than 200 nucleotides. They not only participate in biological processes but also closely link to the metastasis and proliferation of cancer (Zhang et al., 2014). To date, many studies have investigated the functional relationship of lncRNAs and TNBC. For example, Tang et al. (2018) confirmed that lncRNA PVT1 drives the tumorigenesis of TNBC by promoting KLF5/β-catenin signal transduction. And Wang S. et al. (2018) have found that lncRNA MIR100HG promotes TNBC cell proliferation by binding to p27. In addition, some lncRNAs have been revealed to inhibit the tumorigenesis of TNBC. For example, lncRNA RMST can significantly inhibit TNBC cell proliferation, invasion, and migration while promoting apoptosis, suggesting the antitumor effect (Wang L. et al., 2018). LncRNA GHET1 has been proved to be a key regulatory molecule in the development of many cancers. Ni et al. (2018) reported that knockdown of the expression of lncRNA GHET1 can increase the expression of Numb protein, thereby inhibiting the cell activity of the glioma. Prostate cancer cell proliferation is also enhanced by lncRNA GHET1, which functions through increasing the HIF-1α/Notch-1 signaling activity by binding to KLF2 (Zhu et al., 2019). Notably, Han et al. (2019) found that the inhibition of lncRNA GHET1 can downregulate the expression of EGFR protein and inhibit PI3K/AKT signaling activity, thereby inhibiting the cellular activities of breast cancer. Collectively, lncRNA GHET1 has the ability to promote cancer development. But the role and mechanism of lncRNA GHET1 in TNBC progression remain unknown. Therefore, in this study, we characterized the biological role of lncRNA GHET1 in TNBC cells under hypoxia so as to provide a novel target in the treatment and diagnosis of TNBC with an effective theoretical basis.

## Materials and Methods

### Tissue Sample Collection

Tumor tissue samples (the TNBC group) and adjacent normal tissues (the normal group) were collected. These samples were from TNBC patients treated in our hospital between January 2017 and June 2019, with informed consent obtained from each patient. In addition, age, gender, blood pressure, tumor stage, depth of invasion, distant metastasis or not, lymphatic metastasis or not, and other pieces of basic information were recorded.

### Cell Culture and Treatment

Cell lines, provided by American Type Culture Collection, included human normal breast epithelial cell line MCF10A and TNBC cell lines MDA-MB-231, MDA-MB-468, HCC1937, and BT-549. A total of three kinds of media were employed for cell culture, and each medium had proportional supplement of 100 mg/mL penicillin and 10 mg/mL streptomycin (Gibco, United States) and 10% fetal bovine serum (FBS, Gibco, United States). An F12/DMEM 1:1 medium was used for the culture of MCF10A, an L-15 medium for MDA-MB-231 and MDA-MB-468, and a DMEM medium for HCC1937 and BT-549. There were two cell culture environments: normoxia and hypoxia. The former was to culture the cells at 37°C with 5% CO_2_ in normoxic conditions (Normoxia), and the latter was to manipulate the gas content to 94% N_2_, 5% CO_2_, and 1% O_2_ (Hypoxia).

On the completion of culture for 24 h, negative shRNA (sh-NC) or lncRNA GHET1 shRNA interference plasmid (sh-GHET1) was transfected into HCC1937 and MDA-MB-468 cells according to the instructions of Lipofectamine 2000 (Thermo Fisher Scientific, United States). After that, the cells were cultured under Normoxia or Hypoxia condition, named as Normoxia + sh-NC, Normoxia + sh-GHET1, Hypoxia + sh-NC, and Hypoxia + sh-GHET1 groups, respectively. In addition, HCC1937 cells were transfected with an overexpression empty vector (Vector) and an lncRNA GHET1 overexpression vector (GHET1), respectively, for subsequent tumorigenesis in nude mice.

### qRT-PCR

The TRizol method was adopted to extract the total RNA from clinical tissue samples and each cell line, followed by the detection of concentration and purity of RNA by NanoDrop, as well as the preparation of cDNA using a random primer reverse transcription kit (Thermo Fisher Scientific, United States). The SYBRGREEN kit (TaKaRa, Japan) was employed in the determination of lncRNA GHET1 expression, with GAPDH as an internal reference control. The experiment was performed in six replicates. Relative quantification of target gene expression by the 2^–ΔΔ^ ^Ct^ method was based on the experimental data obtained by qRT-PCR. Table 1 presents the primer sequences.

**TABLE 1:** Primer sequences.

RNA	Sequences (5′–3′)
lncRNA GHET1	F: 5′-CCCCACAAATGAAGACACT-3′
	R: 5′-AGTGGCTGGCATATAACCAACA-3′
GAPDH	F: 5′-TCCCATCACCATCTTCCA-3′
	R: 5′-CATCACGCCACAGTTTTCC-3′

### Analysis of 5-Ethynyl-2’-Deoxyuridine (EdU)

Treated HCC1937 and MDA-MB-468 cells were stained according to the instructions of the EdU staining kit (Thermo Fisher Scientific, United States) after being plated in 24-well plates. After that, the cells were mounted using neutral resin, and 6–10 fields under a fluorescence microscope (FM-600, Shanghai Puda Optical Instruments Co., Ltd.) were randomly selected for observation. Based on the recorded number of positive cells in each field, the Edu labeling rate was determined using the following formula: EdU labeling rate (%) = number of positive cells/(number of positive cells + number of negative cells) × 100%.

### Cell Colony Formation Assay

On the completion of the digestion step by 0.25% trypsin, treated HCC1937 and MDA-MB-468 cells were then resuspended with DMEM and L-15 complete media containing 0.35% agarose. Next, 6-well pates containing agarose were used for cell inoculation (1 × 10^5^ cells/well), with subsequent cell culture under Normoxia or Hypoxia condition. After the colonies were formed, their number was counted following a staining step by 0.1% crystal violet solution and a photographing step under an inverted microscope.

### Transwell Invasion Assay

After 24 h of transfection, 2 × 10^4^ HCC1937, MDA-MB-468 cells were added in a transwell upper chamber coated with Matrigel (Becton, Dickinsonand Company, United States) and the lower chamber with the addition of 700 μL of a medium containing 20% FBS. Subsequent cell culture under Normoxia or Hypoxia condition was performed. On the completion of the 24 h culture, the transwell inserts were removed and rinsed twice with PBS. After that, 25 min fixation by 1% glutaraldehyde was first carried out for the invasive cells, followed by a rinsing step by PBS and a drying step. Subsequently, 12 h staining by 0.1% crystal violet was performed, followed by a rinsing step by PBS and a drying step. Then, under an upright microscope, the number of positive cells in random 6–10 fields was observed and recorded. They were randomly selected for observation and determination of the number of positive cells. Finally, three of the fields were photographed for further statistical analysis.

### Detection of Glucose Consumption and Lactate Production Levels

The supernatants of HCC1937 and MDA-MB-468 cell culture media cultured under Normoxia or Hypoxia condition were collected and placed into 10 mL centrifuge tubes, respectively. Centrifugation (4°C, 800 r/min, 5 min) was subsequently performed to remove floating cells and cell debris. Then the supernatants were collected. The extracellular lactate and intracellular glucose were detected with a L-Lactate colorimetric assay kit (ab65330, Abcam, United Kingdom) and a Glucose assay kit (ab65333, Abcam, United Kingdom) according to the manufacturer’s instructions, respectively.

### Western Blotting

The treated cells were used to extract nuclear Yes-associated protein (YAP) and cytoplasmic YAP protein using the nuclear protein extraction kit and cell proteins using a RIPA buffer. After determining the extracted protein concentration by using a BCA Assay Kit, denaturation of 25 μg of protein was performed by boiling in a sample loading buffer (1×). On completion separation by SDS-PAGE, the protein was blotted onto PVDF membranes, which was followed by a 1-h blocking step using 5% skim milk powder. Subsequently, overnight incubation of the membranes with corresponding primary antibodies was performed at 4°C followed by a rinsing step for three times. Another 1-h incubation of the membranes with secondary antibodies at room temperature was also followed by a rinsing step for three times. Following protein development by a chemiluminescence reagent, a gel imaging system was employed to obtain the images and the ImageJ software for the analysis of gray values of the protein bands. GAPDH was used as the internal reference for the extracted whole cell or cytoplasmic proteins, while LaminB was used as the internal reference for the nuclear proteins, thus calculating the relative protein expression.

### Tumorigenesis in Nude Mice

There were four groups of three BALB/c male nude mice each with age of 4–6 weeks. The HCC1937 cells with knockdown or overexpression of lncRNA GHET1 constructed *in vitro* were subcutaneously injected into the right axilla at a number of 1 × 10^6^, respectively. The mice with the injection were named as sh-NC, sh-GHET1, vector, and GHET1 groups. The tumor size, that is, volume = 0.5 × length × width^2^, was measured weekly from the time of subcutaneous injection. Four weeks later, prior to cervical dislocation in mice, 10% chloral hydrate was injected intraperitoneally for anesthesia. The final weight of the removed tumors was measured. This trial was in accordance with animal ethics with the approval of the ethics committee.

### Statistics

Independent sample *t*-test and one-way analysis of variance were performed using SPSS 25.0. Mean ± standard deviation (SD) was the form to present the results. *P* < 0.05 indicated a significant difference.

## Results

### Upregulation of LncRNA GHET1 in Triple-Negative Breast Cancer and Promotion of LncRNA GHET1 by Hypoxia

**FIGURE 1:**
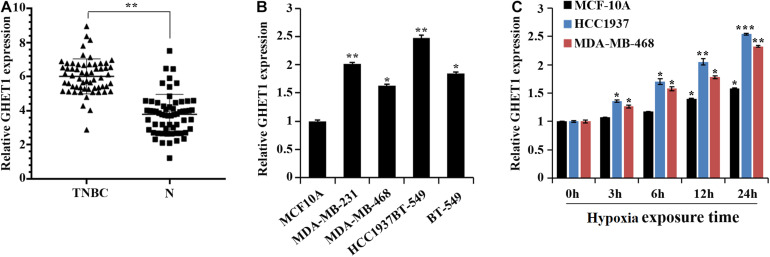
lncRNA GHET1 is upregulated in TNBC and hypoxia promotes the expression of lncRNA GHET1. **(A)** The expression of lncRNA GHET1 in TNBC and adjacent normal tissues was examined by qRT-PCR. **P < 0.01. **(B)** The expression of lncRNA GHET1 was measured in human normal breast epithelial cell MCF10A and TNBC cell lines MDA-MB-231, MDA-MB-468, HCC1937, and BT-549 using qRT-PCR. *P < 0.05 and **P < 0.01 vs. MCF10A. **(C)** MCF10A, MDA-MB-468, and HCC1937 cells were cultured under hypoxia conditions for 3, 6, 12, and 24 h. The expression of lncRNA GHET1 was analyzed by qRT-PCR. *P < 0.05 and **P < 0.01 vs. 0 h. TNBC, triple-negative breast cancer.

At the tissue level, a marked increase in the GHET1 expression in the TNBC tissues was revealed compared to the adjacent normal tissues (Figure 1A). At the cellular level, the GHET1 expression in the TNBC cells was significantly higher than that in the MCF10A cells (Figure 1B). MDA-MB-468 with the relative lowest GHET1 expression and HCC1937 with the highest one were used for subsequent experiments to ensure the study reliability. In addition, with the increase in hypoxia exposure time, GHET1 expression was significantly increased in HCC1937 and MDA-MB-468 cells (Figure 1C).

### Inhibition of Hypoxia-Induced Proliferation, Invasion, and Glycolysis of Triple-Negative Breast Cancer Cells by Downregulation of LncRNA GHET1

**FIGURE 2:**
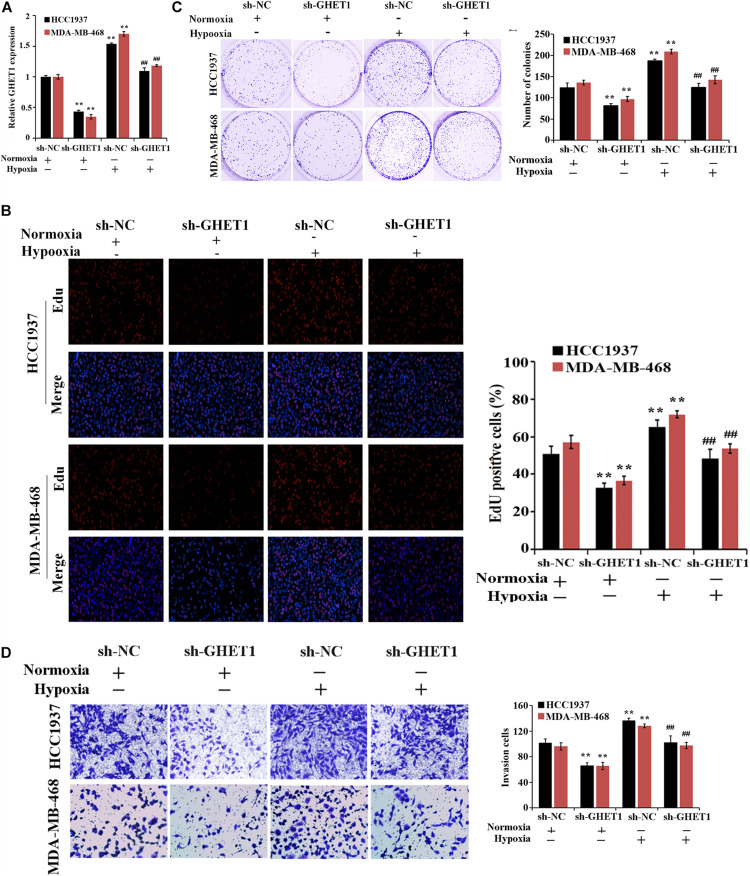
Inhibition of hypoxia-induced proliferation and invasion of TNBC cells by downregulation of lncRNA GHET1. **(A)** qRT-PCR was performed to detect the expression of lncRNA GHET1 in MDA-MB-468 and HCC1937 cells under normoxia and hypoxia conditions. **(B)** EdU assay wad used to detect the cell proliferation. **(C)** Cell viability was analyzed by colony formation assay. **(D)** Transwell assay was used to detect cell invasion. **P < 0.01 vs. Normoxia + sh-NC group; ^##^*P* < 0.01 vs. Hypoxia + sh-NC group.

For the purpose of determination of the lncRNA GHET1 function, lncRNA GHET1 was knocked down *in vitro* and transfected into HCC1937 and MDA-MB-468 cells. The qRT-PCR experiment verified the transfection efficiency. Hypoxia could significantly promote the expression of GHET1 in the cells. At the same time, sh-GHET1 significantly reduced the expression of lncRNA GHET1 in the cells under hypoxia and hypoxia conditions (Figure 2A).

**FIGURE 3:**
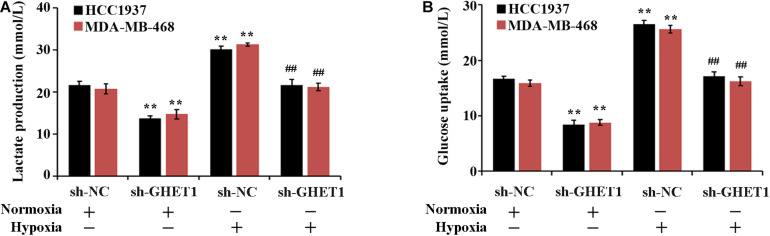
Low expression of lncRNA GHET1 can inhibit hypoxia-induced glycolysis o f TNBC cells. MDA-MB-468 and HCC1937 cells were transfected with sh-GHET1 or control plasmid under normoxia and hypoxia conditions. The lactate production **(A)** and glucose uptake **(B)** were measured. **P < 0.01 vs. Normoxia + sh-NC group; ^##^*P* < 0.01 vs. Hypoxia + sh-NC group.

The effect of lncRNA GHET1 on the TNBC cell function was further examined. Under normoxic conditions, after interfering with the expression of lncRNA-GHET1, the cell proliferation (Figure 2B), viability (Figure 2C), invasion (Figure 2D), lactate production (Figure 3A), and lactate glucose consumption (Figure 3B) of HCC1937 and MDA-MB-468 cells were significantly inhibited. However, when the cells were under hypoxic conditions, the above indicators in the cells were significantly increased (*p* < 0.05). And compared with the Hypoxia + sh-NC group, the cell indicators of the Hypoxia + sh-GHET1 group were significantly reduced (*p* < 0.05). Together, these results provided evidence that hypoxia-induced proliferation, invasion, and glycolysis of TNBC cells could be inhibited by the downregulation of lncRNA GHET1.

### Inhibition of Hypoxia-Induced Hippo/YAP Pathway Activation and YAP Nuclear Translocation by Downregulation of LncRNA GHET1

**FIGURE 4:**
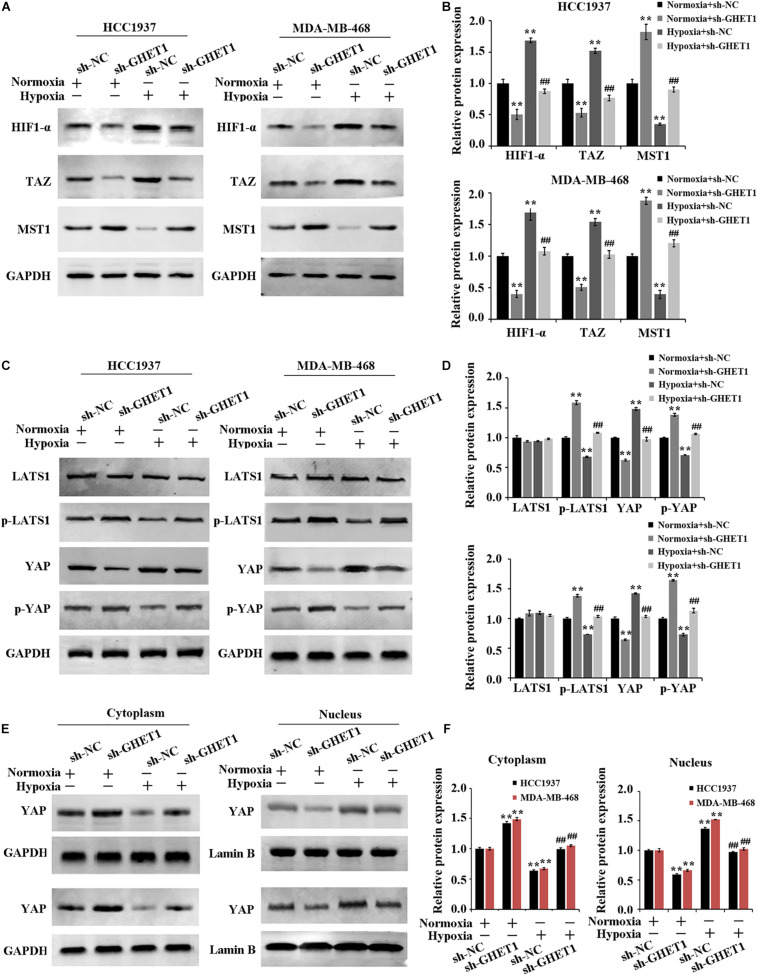
Inhibition of hypoxia-induced Hippo/YAP pathway activation and YAP nuclear translocation by downregulation of lncRNA GHET1 MDA-MB-468 and HCC1937 cells were transfected with sh-GHET1 or control plasmid under normoxia and hypoxia conditions. **(A–D)** Protein expression of HIF-α and the indicated Hippo/Yap pathway were measured by western blot. **(E,F)** Expression of YAP protein in the cytoplasm and nucleus of TNBC cells was detected by western blot. **P < 0.01 vs. Normoxia + sh-NC group; ^##^*P* < 0.01 vs. Hypoxia + sh-NC group.

The expression of Hippo/YAP pathway-related proteins was detected to further determine the molecular mechanism of lncRNA GHET1 regulating TNBC cell function. The results in Figure 3A showed that the expression of HIF-1α was significantly upregulated under hypoxia conditions. After interfering with the expression of lncRNA GHET1, the expression of HIF-1α was significantly downregulated. In addition, western blotting results (Figures 4A–F) revealed that compared with normoxia conditions, the protein expression of MAT1, p-LATS1, p-YAP, and YAP (cytoplasm) was decreased, while the expression of TAZ, YAP (cells), and YAP (nucleus) was increased in the HCC1937 cells and MDA-MB-468 cells under hypoxia conditions. However, after knockdown of lncRNA GHET1, a significant increase in the protein expression of MAT1, p-LATS1, p-YAP, and YAP (cytoplasm), and a significant decrease in the expression of TAZ, YAP (cells), and YAP (nucleus), were found compared to the groups with transfection of sh-NC under normoxia or hypoxia conditions.

These results determined that knockdown of lncRNA GHET1 expression inhibited the expression of HIF-1α, Hippo/YAP pathway activation, and YAP nuclear translocation in TNBC cells in a hypoxia environment.

### *In vivo* Growth Promotion of Triple-Negative Breast Cancer by LncRNA GHET1

**FIGURE 5:**
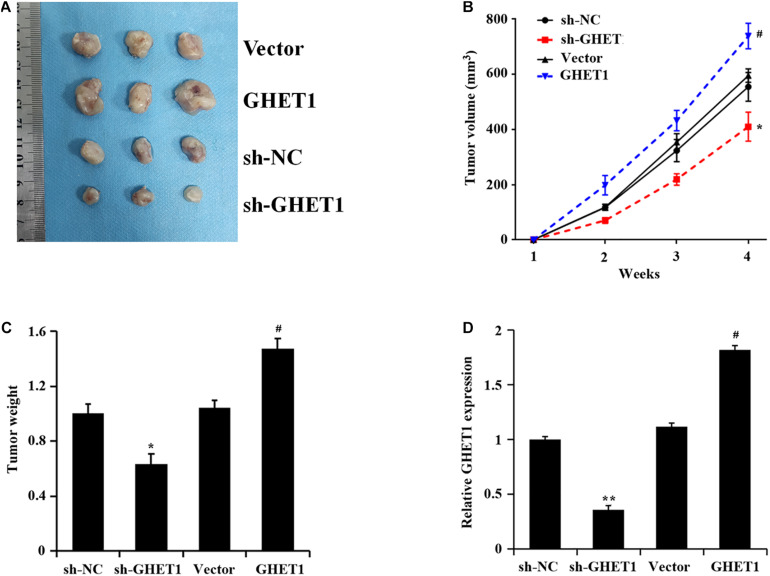
lncRNA GHET1 promoted the tumor growth in nude mice. **(A)** Typical TNBC tumor from tumor bearing mice after subcutaneous injection of HCC1937 cells stably overexpression and underexpression lncRNA GHET. **(B)** The TNBC tumor volume from tumor bearing mice after treatment. **(C)** The tumor weight from tumor bearing mice after treatment. **(D)** Expression of lncRNA GHET1 in xenograft tumor tissues as determined by qRT-PCR. *p < 0.05 and **p < 0.01 vs. sh-NC group; ^#^*p* < 0.05 and ^##^*p* < 0.01 vs. Vector group.

The results of tumorigenesis in nude mice 4 weeks later and at the other time points showed markedly decreased tumor volume and weight after knockdown of lncRNA GHET1, while the opposite change occurred after the overexpression of lncRNA GHET1 (Figures 5A–C). A marked reduction of lncRNA GHET1 in tumor tissues after knockdown of lncRNA GHET1 and a markedly increased one after overexpression of lncRNA GHET1 were confirmed in the qRT-PCR results (Figure 5D).

### Promotion of *in vivo* Hippo/YAP Pathway Activation and YAP Nuclear Translocation in Triple-Negative Breast Cancer by LncRNA GHET1

**FIGURE 6:**
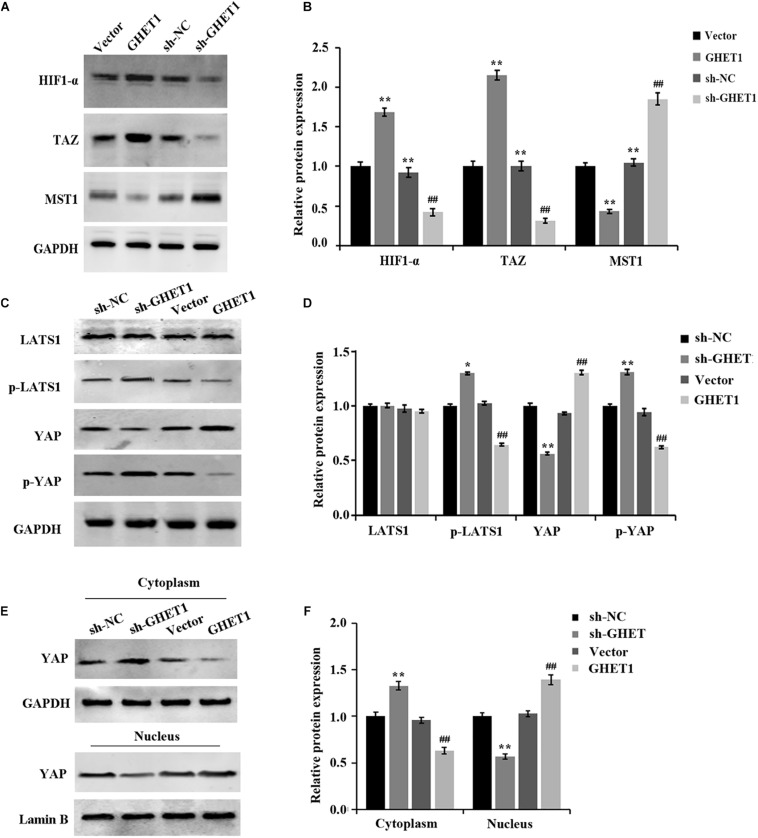
Promotion of *in vivo* Hippo/YAP pathway activation and YAP nuclear translocation in triple-negative breast cancer by lncRNA GHET1. **(A–D)** Proteins expression of HIF-α and the indicated Hippo/Yap pathway in xenograft tumor tissues was detected by western blot. **(E,F)** Expression of YAP in the cytoplasm and the nucleus of the xenograft tumor tissues was analyzed using western blot. *p < 0.05 and **p < 0.01 vs. sh-NC group; ^#^*p* < 0.05 and ^##^*p* < 0.01 vs. Vector group.

The results obtained from western blotting were shown in Figures 6A–F. The markedly increased protein expression of MST1, p-LATS1, p-YAP, and YAP (cytoplasm) and the significantly decreased expression of HIF-1α, TAZ, YAP (cells), and YAP (nucleus) in the tumor tissues of the sh-GHET1 group were compared with those of the sh-NC group. However, with the overexpression of lncRNA GHET1, the above protein expression was opposite. These results confirmed that lncRNA GHET1 inhibited the progression of TNBC *in vivo* by decreasing the activation of the Hippo/YAP signaling pathway.

## Discussion

With the deepening of cancer research, many new endocrine drugs for the treatment of TNBC have been developed. However, in terms of individualized treatment, only conventional non-specific cytotoxic drugs can be used and without effective and practical treatment methods. In recent years, the literature on lncRNAs has revealed them as signaling molecules for gene expression regulators and as pivotal factors for tumor development (Luo et al., 2017). In our study, significantly increased lncRNA GHET1 in TNBC tissues and cells was found, and lncRNA GHET1 expression was time-dependent in a hypoxia environment. Therefore, the indication of correlation between lncRNA GHET1 and TNBC cell development can be set out. In addition, hypoxia is also reported as an important external factor that promotes drug resistance and radiation resistance and improves the invasive ability of cancer cells (Samanta et al., 2014). It has been pointed out that the high activity of HIF-1α and its target gene products in TNBC is the key to induce the enhancement of TNBC cell viability, while the inhibition of HIF-1α expression by drugs can bring an effective improved survival rate (Samanta et al., 2014). According to our experimental results, after knockdown of lncRNA GHET1 in HCC1937 and MDA-MB-468 cells, cell proliferation, viability, invasion ability, glucose consumption, lactate production, and the expression of HIF-1α were significantly inhibited under both normoxia and hypoxia conditions. In addition, all abilities of TNBC cells under hypoxia conditions were significantly higher than those under normoxia conditions. These results indicate the possible relationship between the role of lncRNA GHET1 and the promotion of TNBC development, and the expression of lncRNA GHET1 will be induced by hypoxia. In order to further demonstrate the functional relationship between lncRNA GHET1 and TNBC development, tumorigenesis in nude mice was carried out for subsequent *in vivo* experiments. And the results showed that knockdown of lncRNA GHET1 expression significantly reduced the tumor volume and weight, while overexpression of this gene promoted the tumor development. Collectively, lncRNA GHET1 is associated with the tumor development, and the specific mechanism has also been investigated in our study.

More and more studies have confirmed that the Hippo/YAP signaling pathway plays a central role in regulating many aspects of tumor biology (Yu et al., 2015; Zanconato et al., 2016). Studies have shown that the abnormality of this signal has been proved to be the key to the development of breast cancer, lung cancer, and renal cancer (Tordjmann, 2011; Zhao et al., 2011), while its degree of abnormality is often closely related to the prognosis of tumors. When the Hippo/YAP signaling pathway is activated, MST1 kinase activates the LATS protein. The LATS protein receives signal phosphorylation, transcription activates TAZ and YAP proteins, and the phosphorylated YAP protein is retained within the cytoplasm, which is subsequently degraded by ubiquitinase (Steinhardt et al., 2008; Janse van Rensburg et al., 2018). YAP, an effector of the Hippo/YAP signaling pathway, is an oncoprotein, and its abnormal activation is an important driver of tumorigenesis, chemoresistance, and metastasis (Xu et al., 2009). In addition, YAP regulates the expression of downstream genes by entering the nucleus and binding to the transcription factor TEAD, thereby mediating the proliferation and malignant transformation of cells (Zhao et al., 2010). It has been shown that the industrial chemical bisphenol promotes YAP nuclear accumulation and upregulates CTGF and ANKRD1 by inhibiting the phosphorylation of YAP, thereby inducing TNBC cell migration (Deng et al., 2018). Collectively, the Hippo/YAP signaling pathway has a close link with TNBC development. In the present study, hypoxia would promote the nuclear transfer of YAP and regulates the expression of key proteins in the Hippo/YAP signaling pathway. After the knockdown of the lncRNA GHET1 expression, phosphorylation of LATS and YAP proteins is promoted and degradation of YAP is induced; thus, the oncogenic activity is inhibited in both cells in a hypoxia environment and tumors *in vivo*. Overexpression of lncRNA GHET1, on the other hand, induces aberrant activation of Hippo/YAP signaling.

## Conclusion

In summary, the hypoxia-induced increase in the GHET1 expression is involved in TNBC progression. And the knockdown of the GHET1 expression can inhibit the expression of HIF-1α and the activation of the Hippo/YAP signaling pathway, thereby reducing the biological activities of TNBC cells such as proliferation and invasion. Therefore, the knockdown of GHET1 is a new way to treat TNBC. However, the exact mechanism by which lncRNA GHET1 stabilizes the activity of the Hippo/YAP signaling pathway is not clear. Further research should be conducted for exploring the biological function of lncRNA GHET1.

## Data Availability Statement

The original contributions presented in the study are included in the article/supplementary material, further inquiries can be directed to the corresponding author/s.

## Ethics Statement

The studies involving human participants were reviewed and approved by the Medical Research Ethics Committee of The First Affiliated Hospital of Jinzhou Medical University. Written informed consent for participation was not required for this study in accordance with the national legislation and the institutional requirements.

## Author Contributions

YW and SL: study concept and design, acquisition of data, analysis and interpretation of data, drafting of the manuscript, critical revision of the manuscript for important intellectual content, statistical analysis, administrative, technical and material support, and study supervision. Both authors have read and approved the manuscript.

## Conflict of Interest

The authors declare that the research was conducted in the absence of any commercial or financial relationships that could be construed as a potential conflict of interest.

## References

[B1] Biswas S. Rao C. M. (2017). Epigenetics in cancer: fundamentals and beyond. *Pharmacol. Therapeutics.* 173 118–134. 10.1016/j.pharmthera.2017.02.011 28188812

[B2] Cesi G. Walbrecq G. Zimmer A. Kreis S. Haan C. (2017). Ros production induced by braf inhibitor treatment rewires metabolic processes affecting cell growth of melanoma cells. *Mol. Cancer.* 16:102. 10.1186/s12943-017-0667-y PMC5465587 28595656

[B3] Deng Q. Jiang G. Wu Y. Li J. Liang W. Chen L. (2018). Gper/hippo-yap signal is involved in bisphenol s induced migration of triple negative breast cancer (tnbc) cells. *J. Hazardous Mat.* 355 1–9. 10.1016/j.jhazmat.2018.05.013 29758456

[B4] Fan L. Strasser-Weippl K. Li J. J. St Louis J. Finkelstein D. M. Yu K. D. (2014). Breast cancer in china. *Lancet Oncol.* 15 e279–e289. 24872111 10.1016/S1470-2045(13)70567-9

[B5] Haffty B. G. Yang Q. Reiss M. Kearney T. Higgins S. A. Weidhaas J. (2006). Locoregional relapse and distant metastasis in conservatively managed triple negative early-stage breast cancer. *J. Clin. Oncol.* 24 5652–5657. 10.1200/jco.2006.06.5664 17116942

[B6] Han M. Wang Y. Gu Y. Ge X. Seng J. Guo G. (2019). Lncrna ghet1 knockdown suppresses breast cancer activity in vitro and in vivo. *Am. J. Transl. Res.* 11 31–44. 30787968 PMC6357318

[B7] Huang R. Zong X. (2017). Aberrant cancer metabolism in epithelial-mesenchymal transition and cancer metastasis: mechanisms in cancer progression. *Critical Rev. Oncol. Hematol.* 115 13–22. 10.1016/j.critrevonc.2017.04.005 28602165

[B8] Janse van Rensburg H. J. Azad T. Ling M. Hao Y. Snetsinger B. Khanal P. (2018). The hippo pathway component taz promotes immune evasion in human cancer through pd-l1. *Cancer Res.* 78 1457–1470. 10.1158/0008-5472.can-17-3139 29339539

[B9] Luo H. Yang H. Lin Y. Zhang Y. Pan C. Feng P. (2017). Lncrna and mrna profiling during activation of tilapia macrophages by hsp70 and streptococcus agalactiae antigen. *Oncotarget* 8 98455–98470. 10.18632/oncotarget.21427 29228702 PMC5716742

[B10] Manoochehri Khoshinani H. Afshar S. Najafi R. (2016). Hypoxia: a double-edged sword in cancer therapy. *Cancer Invest.* 34 536–545. 10.1080/07357907.2016.1245317 27824512

[B11] Ni W. Luo L. Zuo P. Li R. P. Xu X. B. Wen F. (2018). Lncrna ghet1 down-regulation suppresses the cell activities of glioma. *Cancer Biomarkers Sec. A Dis. Markers.* 23 9–22. 10.3233/cbm-171002 30103301 PMC13078550

[B12] Samanta D. Gilkes D. M. Chaturvedi P. Xiang L. Semenza G. L. (2014). Hypoxia-inducible factors are required for chemotherapy resistance of breast cancer stem cells. *Proc. Natl. Acad. Sci. U S A.* 111 E5429–E5438. 25453096 10.1073/pnas.1421438111 PMC4273385

[B13] Semenza G. L. (2012). Hypoxia-inducible factors: mediators of cancer progression and targets for cancer therapy. *Trends Pharmacol. Sci.* 33 207–214. 10.1016/j.tips.2012.01.005 22398146 PMC3437546

[B14] Siegel R. L. Miller K. D. Jemal A. (2019). Cancer statistics, 2019. *CA Cancer J. Clin.* 69 7–34. 30620402 10.3322/caac.21551

[B15] Singh D. Arora R. Kaur P. Singh B. Mannan R. Arora S. (2017). Overexpression of hypoxia-inducible factor and metabolic pathways: possible targets of cancer. *Cell Biosci.* 7:62. 10.1186/s13578-017-0190-2 PMC5683220 29158891

[B16] Steinhardt A. A. Gayyed M. F. Klein A. P. Dong J. Maitra A. Pan D. (2008). Expression of yes-associated protein in common solid tumors. *Hum. Pathol.* 39 1582–1589. 10.1016/j.humpath.2008.04.012 18703216 PMC2720436

[B17] Tang J. Li Y. Sang Y. Yu B. Lv D. Zhang W. (2018). Lncrna pvt1 regulates triple-negative breast cancer through klf5/beta-catenin signaling. *Oncogene* 37 4723–4734. 10.1038/s41388-018-0310-4 29760406

[B18] Tordjmann T. (2011). Hippo signalling: liver size regulation and beyond. *Clin. Res. Hepatol. Gastroenterol.* 35 344–346. 10.1016/j.clinre.2011.01.012 21377440

[B19] Wang L. Liu D. Wu X. Zeng Y. Li L. Hou Y. (2018). Long non-coding rna (lncrna) rmst in triple-negative breast cancer (tnbc): expression analysis and biological roles research. *J. Cell. Physiol.* 233 6603–6612. 10.1002/jcp.26311 29215701

[B20] Wang S. Ke H. Zhang H. Ma Y. Ao L. Zou L. (2018). Lncrna mir100hg promotes cell proliferation in triple-negative breast cancer through triplex formation with p27 loci. *Cell death & disease.* 9 805. 10.1038/s41419-018-0869-2 PMC6057987 30042378

[B21] Xu M. Z. Yao T. J. Lee N. P. Ng I. O. Chan Y. T. Zender L. (2009). Yes-associated protein is an independent prognostic marker in hepatocellular carcinoma. *Cancer* 115 4576–4585. 10.1002/cncr.24495 19551889 PMC2811690

[B22] Yu F. X. Zhao B. Guan K. L. (2015). Hippo pathway in organ size control, tissue homeostasis, and cancer. *Cell* 163 811–828. 10.1016/j.cell.2015.10.044 26544935 PMC4638384

[B23] Zanconato F. Cordenonsi M. Piccolo S. (2016). Yap/taz at the roots of cancer. *Cancer Cell.* 29 783–803. 10.1016/j.ccell.2016.05.005 27300434 PMC6186419

[B24] Zhang A. Xu M. Mo Y. Y. (2014). Role of the lncrna-p53 regulatory network in cancer. *J. Mol. Cell Biol.* 6 181–191. 10.1093/jmcb/mju013 24721780 PMC4034727

[B25] Zhao B. Li L. Tumaneng K. Wang C. Y. Guan K. L. (2010). A coordinated phosphorylation by lats and ck1 regulates yap stability through scf(beta-trcp). *Genes Dev.* 24 72–85. 10.1101/gad.1843810 20048001 PMC2802193

[B26] Zhao B. Tumaneng K. Guan K. L. (2011). The hippo pathway in organ size control, tissue regeneration and stem cell self-renewal. *Nat. Cell. Biol.* 13 877–883. 10.1038/ncb2303 21808241 PMC3987945

[B27] Zhu Y. Tong Y. Wu J. Liu Y. Zhao M. (2019). Knockdown of lncrna ghet1 suppresses prostate cancer cell proliferation by inhibiting hif-1α/notch-1 signaling pathway via klf2. *BioFactors* 45 364–373. 10.1002/biof.1486 30609158

